# Emerging treatment approaches for VEXAS syndrome: a systematic review and meta-analysis

**DOI:** 10.1007/s00277-025-06382-2

**Published:** 2025-04-27

**Authors:** Berkay Kilic, Efe Sacin, Muhammet Kadir Tanin, Ozgur Can Kilinc, Serdal Ugurlu

**Affiliations:** 1 Cerrahpasa Medical Faculty, Istanbul University-Cerrahpasa, Istanbul, Turkey; 2 Division of Rheumatology, Department of Internal Medicine, Cerrahpasa Medical Faculty, Istanbul University-Cerrahpasa, Istanbul, 3409 Turkey

**Keywords:** VEXAS, Myelodyplastic syndrome, Autoinflammatory diseases, Azacitidine, JAK inhibitors, Biological agents

## Abstract

VEXAS (vacuoles, E1 enzyme, X-linked, autoinflammatory, somatic) syndrome is a monogenic autoinflammatory disorder with significant morbidity and mortality. Numerous treatment options including azacitidine, JAK inhibitors, IL-6 inhibitors, anti-IL-1, and anti-TNF agents have been proposed. However, no consensus on optimal treatment algorithm has been reached. This study aims to evaluate the efficacy and safety of medical treatment options through a meta-analysis of existing data to help establish clearer guidelines for managing VEXAS. The study protocol was registered in PROSPERO (CRD42024590134). MEDLINE and EMBASE were screened from inception until March 2025. We included patients with VEXAS syndrome who received treatment with azacitidine, JAK inhibitors, IL-6 inhibitors, anti-IL-1, or anti-TNF agents. The primary outcome was the proportion of complete responders. Partial response and reported adverse events were also evaluated. A total of 16 studies and 367 patients with VEXAS syndrome were included. Concomitant myelodysplastic syndrome (MDS) was reported in 149 (40.6%) patients. Azacitidine treatment resulted in complete and partial response in 67% [95% CI (0.56,0.77)] and in 73% [95% CI (0.64,0.82)] of cases, respectively. JAK inhibitors produced a complete response in 42% [95% CI (0.33,0.52)] and partial response in 79% [95% CI (0.71,0.87)]. IL-6 inhibitors led to a complete response in 24% [95% CI (0.15,0.32)] and partial response in 72% [95% CI (0.64,0.81)]. Adverse events were frequently observed. Azacitidine demonstrated significant efficacy in patients with MDS. JAK inhibitors and IL-6 inhibitors may also be viable treatment options. Prospective clinical trials are needed for further confirmation of the results.

**Supplementary Information:**

The online version contains supplementary material available at 10.1007/s00277-025-06382-2.

## Introduction

VEXAS (vacuoles, E1 enzyme, X-linked, autoinflammatory, somatic) syndrome is a monogenic autoinflammatory disease predominantly affecting males over the age of 50 [1, 2]. Even though the precise molecular mechanisms leading to VEXAS need elucidation, pathogenic variants in the ubiquitin-like modifier activating enzyme 1 (UBA1) gene have been established as the key etiological factor [3]. These variations in the UBA1 gene primarily affect the myeloid lineage, disrupting the ubiquitination and leading to protein misfolding, which results in excessive inflammation and bone marrow failure [4].

The clinical presentation of VEXAS is broad and overlaps significantly with various rheumatologic and hematologic disorders, which often leads to misdiagnosis. Common manifestations include skin lesions, fever, weight loss, lung involvement, chondritis, arthralgia, fatigue, cytopenia, myelodysplastic syndrome (MDS), thrombosis, and hematologic malignancies [2, 5, 6]. Furthermore, the disease is associated with significant mortality and morbidity [7].

Despite ongoing research on various treatment modalities for VEXAS syndrome, researchers were unable to achieve a precise consensus on a treatment algorithm. Among the available therapies, patients tend to respond most profoundly to glucocorticoids, though their use is associated with significant adverse effects and lacks curative potential. The therapeutic goal is to taper the glucocorticoid dosage while maintaining disease control [8]. A relatively invasive intervention with curative potential is allogeneic hematopoietic stem cell transplantation (allo-HSCT) [9]. The remaining treatment options are medical therapies that, while lacking curative effects, have shown promise in alleviating symptoms and reducing glucocorticoid dependence to some extent by targeting either inflammation or clonal cells.

A therapeutic approach involves inhibiting inflammatory signaling through the Janus kinase-signal transducer and activator of transcription (JAK-STAT) pathway, which plays a central role in inflammation. JAK inhibitors such as ruxolitinib, tofacitinib, baricitinib, and upadacitinib have been used in the management of VEXAS syndrome [10]. Tocilizumab and sarilumab, both humanized antibodies targeting interleukin-6 (IL-6), have also been employed in the treatment of VEXAS [11, 12]. Key anti-IL-1 agents used in treating VEXAS and other inflammatory diseases include anakinra and canakinumab, which act as IL-1R antagonists and IL-1β inhibitors, respectively [13]. Anti-tumor necrosis factor (TNF) agents, including infliximab and adalimumab, are also part of the therapeutic landscape [14]. Additionally, targeting the clonal cells harboring UBA1 gene mutations represents another avenue of treatment. Hypomethylating agents, particularly azacitidine, a pyrimidine nucleoside analogue and established chemotherapy agent, have been utilized for this purpose [15].

Nevertheless, evidence regarding the efficacy and safety of these medical therapeutic options remains limited. This systematic review and meta-analysis aim to evaluate the efficacy and safety of these treatments to aid in the development of a standardized treatment algorithm for VEXAS syndrome according to the principles of evidence-based medicine.

## Methods

This systematic review was conducted in accordance with the Preferred Reporting Items for Systematic Reviews and Meta-Analyses (PRISMA) 2020 Statement [16] and the study protocol was registered on The International Prospective Register of Systematic Reviews (PROSPERO) with registration number CRD42024590134.

### Eligibility criteria

Patients diagnosed with VEXAS syndrome, confirmed by pathogenic mutations in the UBA1 gene, were included in this study. The interventions of interest included: azacitidine, JAK inhibitors (ruxolitinib, tofacitinib, baricitinib, upadacitinib), IL-6 inhibitors (tocilizumab, sarilumab), IL-1 inhibitors (anakinra, canakinumab), and anti-TNF agents (infliximab, adalimumab, etanercept). Individuals who had previously received or were concurrently receiving corticosteroids, immunosuppressive agents, or conventional disease-modifying anti-rheumatic drugs were deemed eligible. Eligible study designs for our systematic review were randomized controlled trials (RCTs), non-RCTs, prospective observational studies, retrospective observational studies, and case series involving three or more patients. We did not exclude conference abstracts to increase the comprehensiveness and precision of the systematic review and to decrease the potential risk of publication bias.

Other systemic autoinflammatory diseases and unconfirmed diagnoses were excluded from the study. Patients who had undergone allogeneic hematopoietic stem cell transplantation were also not eligible for inclusion. Treatment with conventional disease-modifying anti-rheumatic drugs was also not analyzed as an intervention of interest. Additionally, studies that did not evaluate the relevant outcome measures, as specified in the “Data Extraction and Outcome Measures” section, studies with fewer than three patients, and review articles lacking original data were excluded from consideration. No restrictions were applied regarding language, country of origin, or institutional setting (i.e., single-center, multicenter) during the study selection process.

### Literature search

A pilot literature search was conducted by S.U. to assess the feasibility of the systematic review. Subsequently, one reviewer (B.K.) conducted a systematic literature search using the medical databases MEDLINE (via PubMed) and EMBASE from inception to March 2025. A single medical subject heading term “VEXAS” was used in the search strategy. In addition, conference abstracts from the last three years of the American College of Rheumatology (ACR), American Society of Hematology (ASH), and The European Alliance of Associations for Rheumatology (EULAR) congresses were screened at their respective websites for eligible studies.

### Study selection and data extraction

The study selection process consisted of three elements: title and abstract screening, full-text screening, and crosscheck. This procedure was carried out by two independent reviewers (B.K. and S.U.). Two independent reviewers (E.S and S.U.) performed data extraction and crosschecked the results. Any disagreements between the reviewers were resolved through discussion and reaching a consensus or by consulting a third reviewer. When reviewers suspected multiple publications from the same cohort (studies conducted in the same region, by the same investigators), they included the most recent publication to avoid including the same patients multiple times. We used Covidence systematic review software (Veritas Health Innovation, Melbourne, Australia) to manage the study selection and data extraction processes.

### Outcome measures

The primary outcome of this study was the proportion of patients who achieved complete response during treatment. Secondary outcomes were the proportion of partial responders and the occurrence of mild or serious adverse events. Given the absence of a standardized consensus on response criteria for VEXAS syndrome, we developed a set of criteria to be utilized in our systematic review. Our selected criteria for complete response were as follows: a clinically asymptomatic state, with inflammatory and hematological laboratory parameters within the normal range, daily prednisolone administration < 10 mg/day, or prednisolone independence, or genetic remission [Variant allele frequency (VAF) < 1%]. Partial response was defined as a markable improvement in symptoms and laboratory parameters, along with a reduction in prednisolone dosage or VAF. The complete and partial response criteria in our study have been established in accordance with those utilized in the largest reported cohorts on this topic, which are the French VEXAS Group (FRENVEX), Japan VEXAS Group, and Autoinflammatory Diseases Alliance (AIDA) VEXAS registry [17–19]. These criteria were applied only in studies where response criteria were not explicitly defined. If a patient was classified as a complete or partial responder by the study authors, their assessment was considered in the first place. The categorization of the adverse events according to their severity was done based on the classifications provided in the included studies. If not classified in the study, events leading to treatment discontinuation and death were considered serious adverse events.

### Risk of bias assessment

Two reviewers (M.K.T and S.U.) independently assessed the methodological quality of the articles by applying the “Quality Assessment Tool for Observational Cohort and Cross-Sectional Studies” and “Quality Assessment Tool for Case Series Studies” developed by the National Institutes of Health and crosschecked the outcomes. Each study was categorized into good, fair, and poor overall quality according to the answers provided to the questions.

### Statistical analysis

The proportions of patients with 95% confidence intervals (CI) were calculated for binary outcomes. We conducted a pairwise meta-analysis using the fixed-effects model with the application of the inverse variance model to calculate the pooled effect sizes of each outcome measure. The proportion of total variability derived from between-study heterogeneity was assessed using the I^2^ statistic, with I^2^ > 75%, I^2^ > 50%, and I^2^ < 50% corresponding to considerable, moderate, and non-significant heterogeneity, respectively. Zero-event studies were included in the meta-analysis with a 0.5 continuity correction [20]. The outcomes of the meta-analysis were visualized in forest plots. We were not able to conduct any test for funnel plot asymmetry to evaluate the risk of publication bias since none of the interventions included in the meta-analysis contained ten or more studies; however, small-study effects were assessed by conducting sensitivity analyses using a random-effects model and comparing them with the outcomes obtained using fixed-effects model [21]. If the pooled effect size obtained by the random-effects model was more beneficial, we reported this situation in the text and conducted a sensitivity analysis using the leave-one-out method by excluding studies that included less than five patients to take the small-study effects into account. The level of significance was set at 0.05 for all evaluations. The quantitative analysis was performed using the statistical software STATA 16.0 (StataCorp LP, College Station, TX, USA).

## Result

### Study selection

**Fig. 1:**
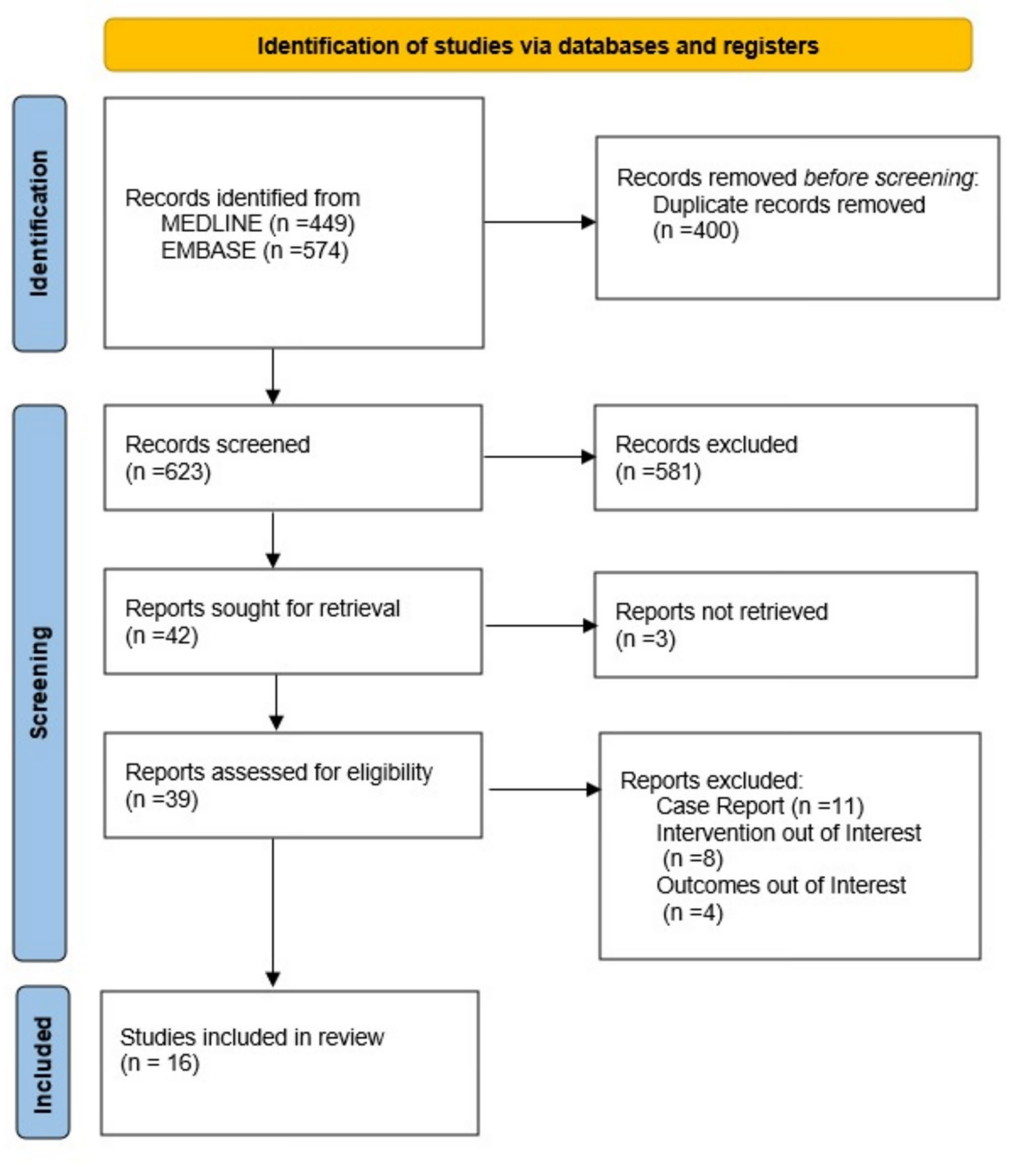
Flowchart describing study selection process

As described in Fig. 1, the initial search yielded 623 records. After title and abstract screening, 39 reports remained for full-text review. Only the most up-to-date versions of the reports from the FRENVEX were selected for review to avoid patient duplication [17, 22]. Eventually, 16 studies that met the inclusion criteria were included in this systematic review and meta-analysis [13, 17–19, 22–33].

### Study characteristics and quality assessment

A total of 367 patients with VEXAS syndrome were included in this study. The included study reports were from 12 countries and published between 2022 and 2025. Among the included patients, 149 (40.6%) were reported to have MDS, while three studies [13, 19, 26] did not provide any information about the number of patients with MDS. Regarding study design,; one study was prospective, seven studies were retrospective cohort, and the rest were case series. Since the response criteria were not defined in the studies conducted by Pinto et al. [27] and Garcia-Escudero et al. [32], these studies were not included in the complete response outcomes. Detailed information about the study characteristics is presented in Table 1. The quality assessments of each study according to the NIH Quality Assessment Tool for Observational Cohort and Case Series Studies are provided in Supplementary Table S1 and Supplementary Table S2.

**Table 1:** Description of the included reports

Study Label	Country	Study design	Article Type	Number of patients, female (*n*)	Number of patients with MDS	Age, median [range]	Indication	Efficacy outcomes	Safety outcomes	Drugs
Hadjadj 2024	France	Retrospective Cohort	Journal Article	110, 1	31	71 [68, 79]	VEXAS	CR, PR	AE, SAE	JAKi, IL-6i, Anti-IL-1, Anti-TNF
Mascaro 2023	Spain	Retrospective Cohort	Journal Article	30, 0	NA	72.9	VEXAS	CR, PR	None	JAKi, Anti-IL-1, IL-6i, Anti-TNF
Campochiaro 2022	Italy	Case Series	Letter	3, 0	NA	69 [68, 70]	VEXAS	CR, PR	AE	Anti-IL-1
Trikha 2024	UK and Northern Ireland	Retrospective Cohort	Letter	4, 0	4	71 [51, 79]	VEXAS with MDS	CR, PR	None	Azacitidine
Pinto 2023	Portugal	Case Series	Letter	3, 0	0	74 [74, 85]	VEXAS	PR	None	IL-6i
Islam 2022	Australia	Case Series	Case Report	3, 0	1	67 [67, 69]	VEXAS	CR, PR	AE, SAE	IL-6i
Johansen 2023	Denmark	Retrospective Cohort	Journal Article	15, 0	4	74 [51, 78]	VEXAS	CR, PR	AE, SAE	IL-6i
Williams 2024	Canada	Case Series	Letter	4, 1	1	65.5 [50, 73]	VEXAS	CR, PR	AE	Azacitidine
Aalbers 2024	Netherlands	Case Series	Letter	6, 0	4	67 [57, 77]	VEXAS	CR, PR	AE	Azacitidine
Kirino 2024	Japan	Prospective Cohort	Journal Article	6, 0	NA	73.5 [55,88]	VEXAS	CR,	AE	IL-6i
Jachiet 2024	France	Retrospective Cohort	Conference Abstract	57, 0	50	71	VEXAS	CR, PR	None	Azacitidine
van der Made 2022	Netherlands	Case Series	Case Report	11, 0	3	67 [47, 79]	VEXAS	CR, PR	AE, SAE	Anti IL-1, IL-6i, Anti-TNF
Salehi 20,223	Australia	Case Series	Brief Report	3, 0	1	72 [69, 72]	VEXAS	CR, PR	AE, SAE	JAKi
García-Escudero 2025	Spain	Retrospective Cohort	Journal Article	39,0	18	72.78 ± 9.23^a^	VEXAS	PR	None	JAKi, IL-6i, Azacitidine, Anti-IL-1, Anti-TNF
Alamo 2025	Spain	Case Series	Journal Article	4,0	0	62 [52–78]	VEXAS without MDS	CR, PR	AE, SAE	Azacitidine
Vitale 2025	Multi-National	Retrospective Cohort	Journal Article	69,4	31	71.8 ± 8^a^	VEXAS	CR, PR	AE, SAE	JAKi, IL-6i, Anti-IL-1, Anti-TNF

^a^ presented in mean ± SD

AE: Adverse events, Anti-IL-1: Anti interleukin-1, Anti-TNF: Anti Tumor Necrosis Factor, CR: Complete response, IL-6i: interleukin-6 inhibitor, JAKi: Janus kinase inhibitor, MDS: Myelodysplastic syndrome, NA: not applicable, PR: Partial response, SAE: Serious Adverse Events, VEXAS: vacuoles, E1 enzyme, X-linked, autoinflammatory, somatic

### Azacitidine

**Fig. 2:**
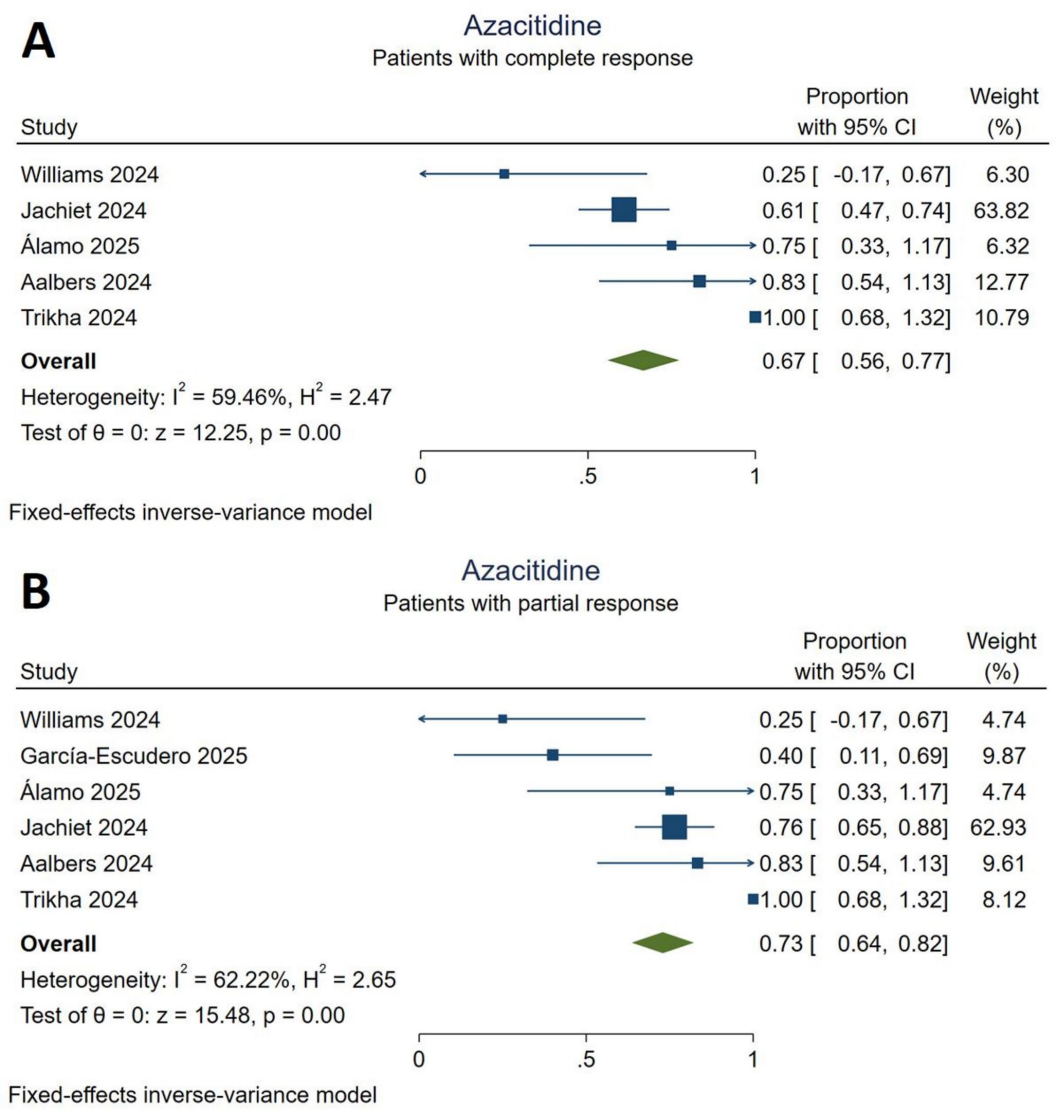
Response to azacitidine treatment. **A**: complete responders, **B**: partial responders

Seventy-nine patients (21.5%) received at least one cycle of azacitidine treatment, at least 59 (74.7%) of whom were reported to have VEXAS-related MDS. The pooled effect sizes indicated that complete and partial was achieved by 67% [95% CI (0.56,0.77)] and 73% [95% CI (0.64,0.82)] of the included patients, respectively (Fig. 2). Moderate heterogeneity was present among the results of complete and partial response outcomes (I^2^ = 59.46% and I^2^ = 62.22%, respectively). A sensitivity analysis conducted using the random-effects model indicated a greater proportion of patients with complete response [70%, 95% CI (0.48,0.91)], providing evidence for the presence of small-study effects (Supplementary Figure S1). Serious infections and related deaths during azacitidine treatment were described in one study [22]. The most commonly observed adverse events associated with azacitidine were infections with nine reported events. Regarding serious adverse events, nine deaths were reported in the included studies (Supplementary Table S3).

### JAK inhibitors

**Fig. 3:**
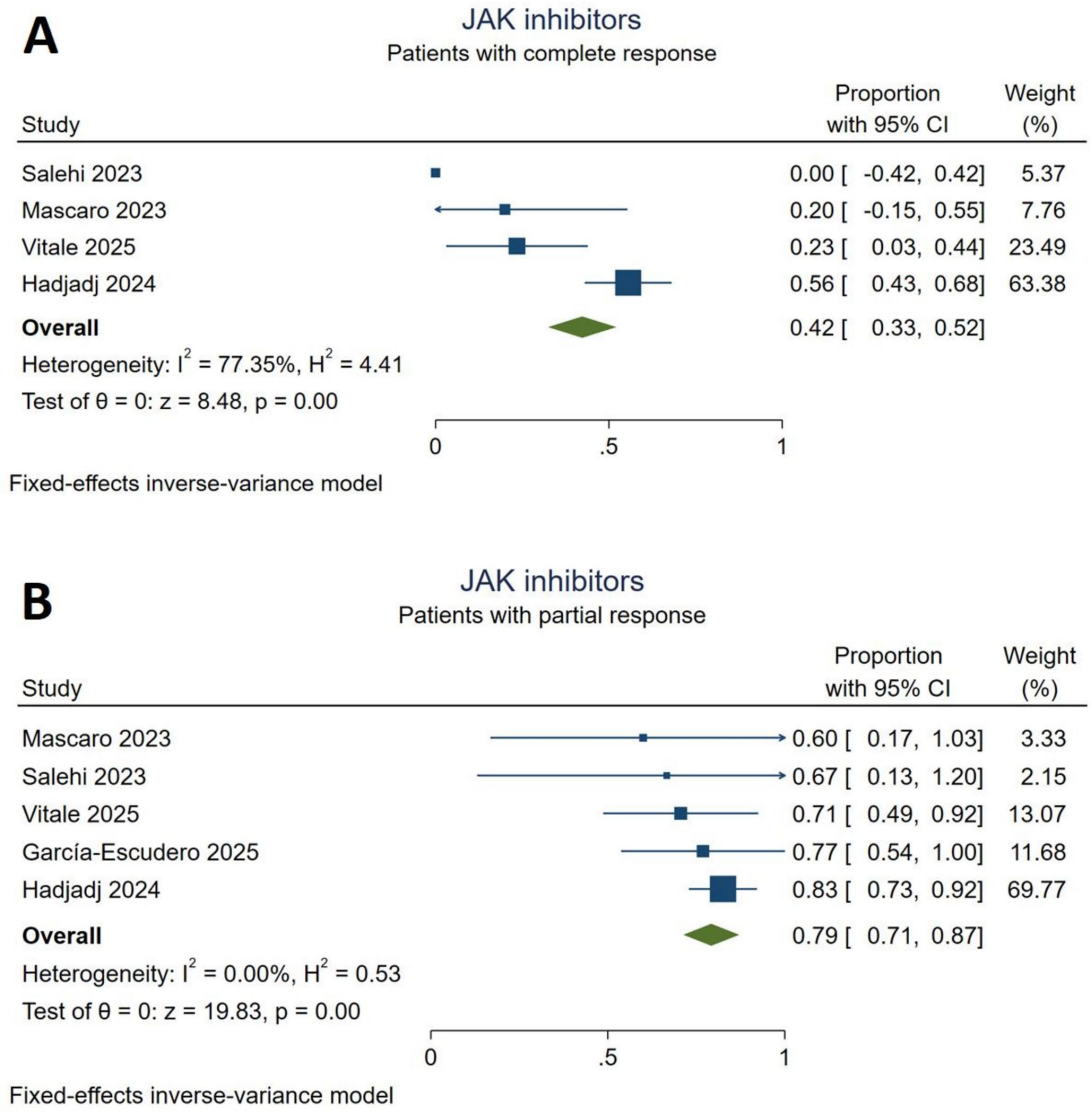
Response to JAK inhibitor treatment. **A**: complete responders, **B**: partial responders

Treatment with JAK inhibitors were administered to 101 (27.5%) patients. The JAK inhibitors that were used in the treatment were ruxolitinib (*n* = 85), tofacitinib (*n* = 15), baricitinib (*n* = 6), upadacitinib (*n* = 3), and filgotinib (*n* = 3). One study [28] included only the patients who received tofacitinib, while four studies [17, 18, 26, 32] reported the overall efficacy and safety outcomes of the administered JAK inhibitors. Since none of these studies reported the specific outcomes for each JAK inhibitor option, we were not able to assess the drugs individually in the quantitative analysis. Outcomes of the meta-analysis indicated that 42% [95% CI (0.33,0.52)] of the patients achieved complete response after treatment with JAK inhibitors. The statistical heterogeneity was considerable for this outcome (I^2^ = 77.35%). JAK inhibitors yielded a partial response in 79% [95% CI (0.71,0.87)] of the included patients with non-significant heterogeneity (I^2^ = 0%, Fig. 3). Cytopenia was the most commonly observed adverse event during JAK inhibitor administration with 20 reported events, followed by infections (nine events) and thrombosis (five events). Cardiovascular events were the most commonly reported serious adverse events during JAK inhibitor treatment (*Supplementary Table S3*).

### IL-6 inhibitors

**Fig. 4:**
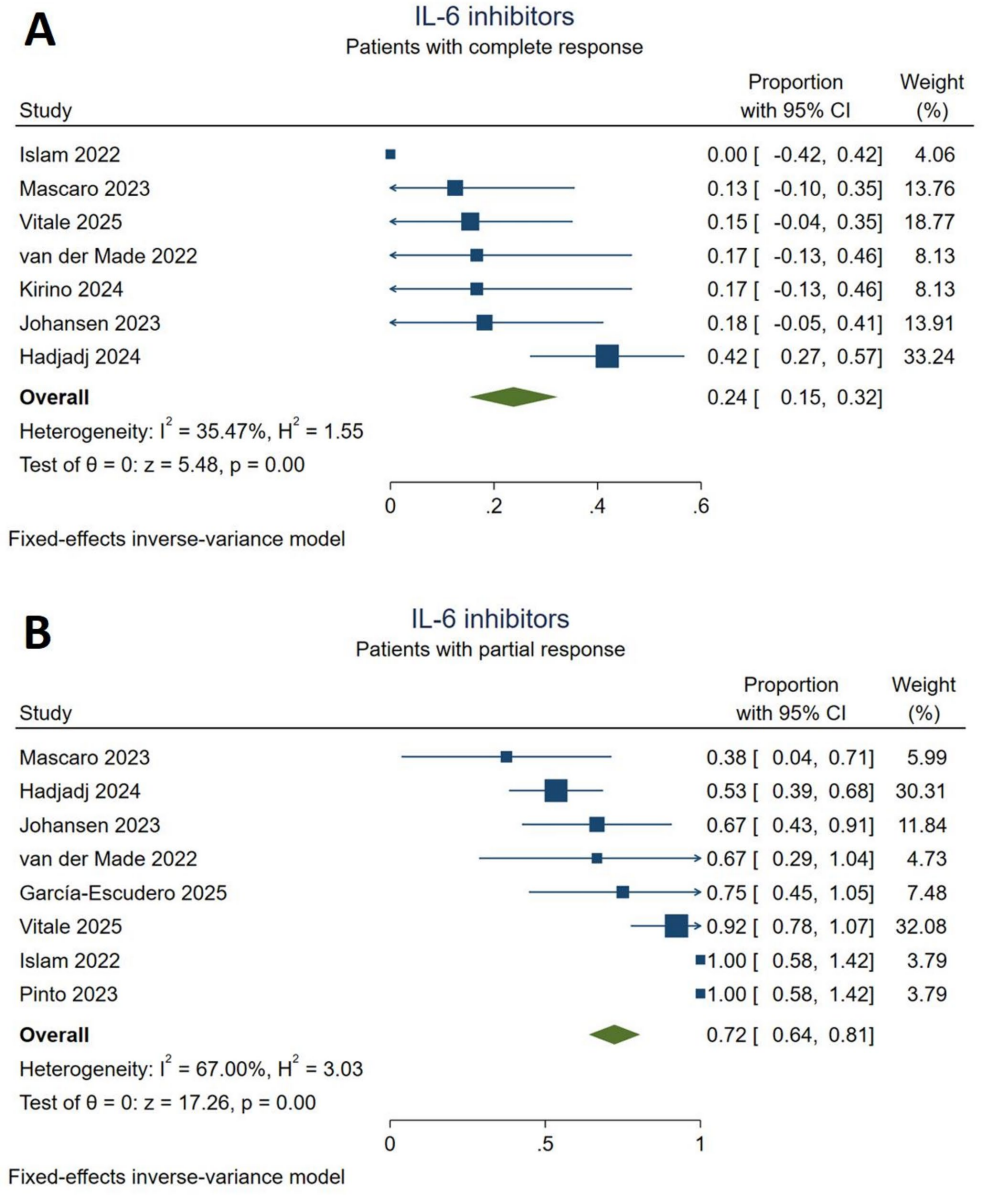
Response to IL-6 inhibitor treatment. **A**: complete responders, **B**: partial responders

One hundred five patients (28.4%) received IL-6 targeting therapy with tocilizumab (*n* = 98), sarilumab (*n* = 7) or both during follow-up. Five studies [19, 24, 27, 30, 32] reported patients who received only tocilizumab and three studies [17, 25, 26] evaluated the outcomes of tocilizumab and sarilumab together. According to the meta-analysis, a complete response was achieved by 24% [95% CI (0.15,0.32)] of the included patients with non-significant statistical heterogeneity (I^2^ = 35.47%). IL-6 inhibitors were associated with a partial response in 72% [95% CI (0.64,0.81)] of the patients, while moderate heterogeneity was present among study results (I^2^ = 67%, Fig. 4). The sensitivity analysis with the random-effects model found a greater proportion of partial responders [74%, 95% CI (0.60,0.89)] to IL-6 inhibitors, indicating possible small-study effects (*Supplementary Figure S2*). To take account of this effect, another sensitivity analysis was conducted using the leave-one-out method by excluding studies with enrollment lower than five patients. In the meta-analysis we conducted by applying this approach, we found that IL-6 inhibitors yielded a partial response in 70% [95% CI (0.61,0.79)] of the patients with moderate statistical heterogeneity (I^2^ = 71.63%, *Supplementary Figure S3*). During treatment with IL-6 inhibitors, 24 cytopenia events were reported, followed by 22 infection events. The most commonly seen serious adverse events were cardiovascular side effects (*Supplementary Table S3).*

### Anti-IL-1 agents

**Fig. 5:**
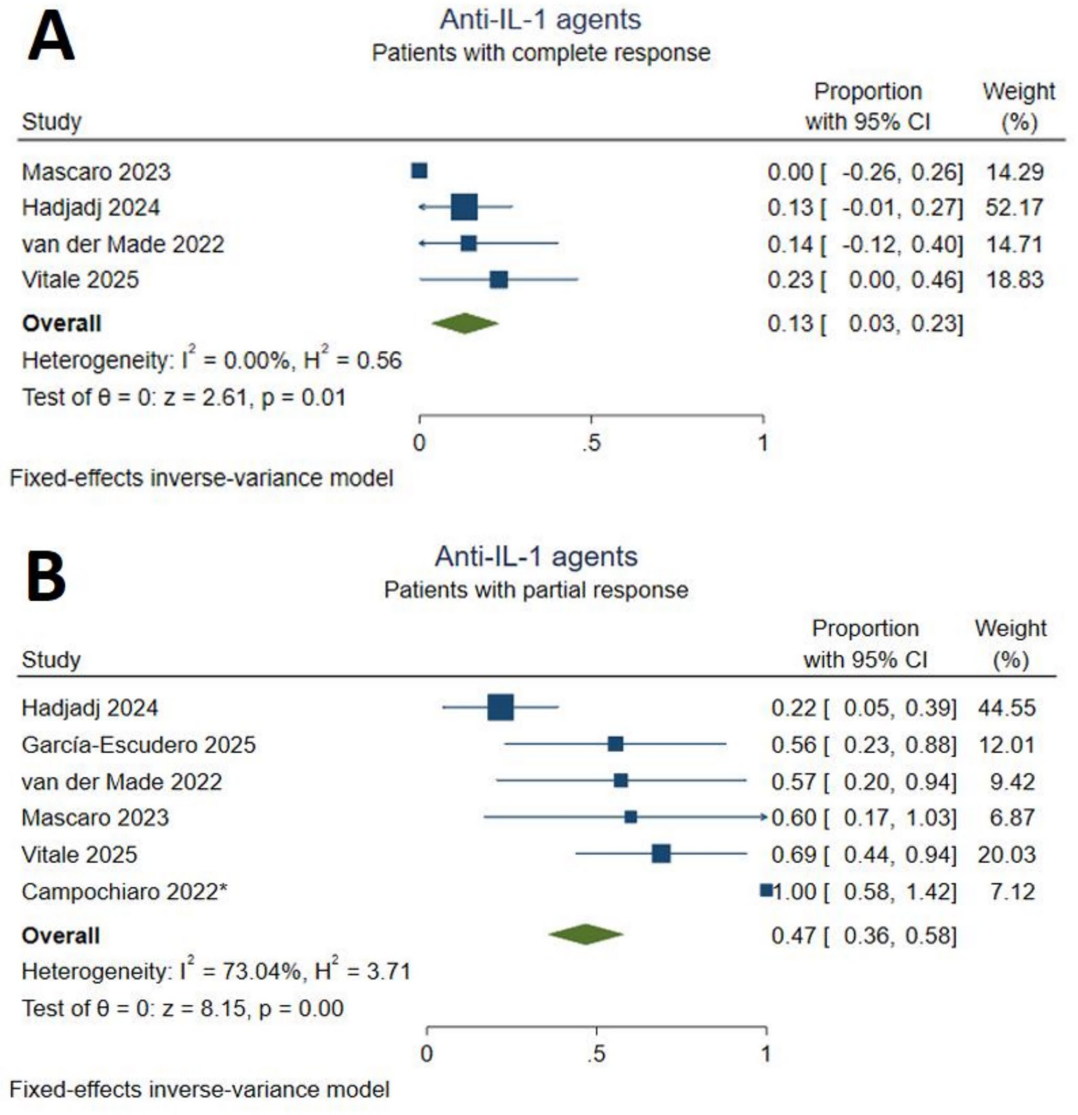
Response to anti-IL-1 treatment. **A**: complete responders, **B**: partial responders

Sixty patients (16.3%) were treated with anti-IL-1 agents, including anakinra (*n* = 41) and canakinumab (*n* = 6). Six studies [13, 17, 18, 26, 30, 32] reported the overall efficacy and safety outcomes of anakinra and canakinumab. Campochiaro et al. [13] administered anti-IL-1 agents in combination with cyclosporin A, which may be a source of methodological heterogeneity. The pooled effect sizes indicated that anti-IL-1 treatment yielded a complete response in 13% [95% CI (0.03,0.13)] of the patients with non-significant statistical heterogeneity (I^2^ = 0%). A partial response to anti-IL-1 treatment was achieved by 47% [95% CI (0.36,0.58)] of the included patients. The statistical heterogeneity was moderate for this outcome (I^2^ = 73.04%, Fig. 5). The sensitivity analysis using the random-effects model indicated a more beneficial result [57%, 95% CI (0.35,0.79)] in the proportion of partial responders, which may provide evidence for small-study effects (*Supplementary Figure S4*). Another sensitivity analysis, which excluded the study by Campochiaro et al. [13] due to the interventional difference, is presented in *Supplementary Figure S5.* Severe injection site reactions (nine events) were the most frequent adverse events of anti-IL-1 agents among included patients. Cytopenia was also commonly reported (*Supplementary Table S3).*

### Anti-TNF agents

**Fig. 6:**
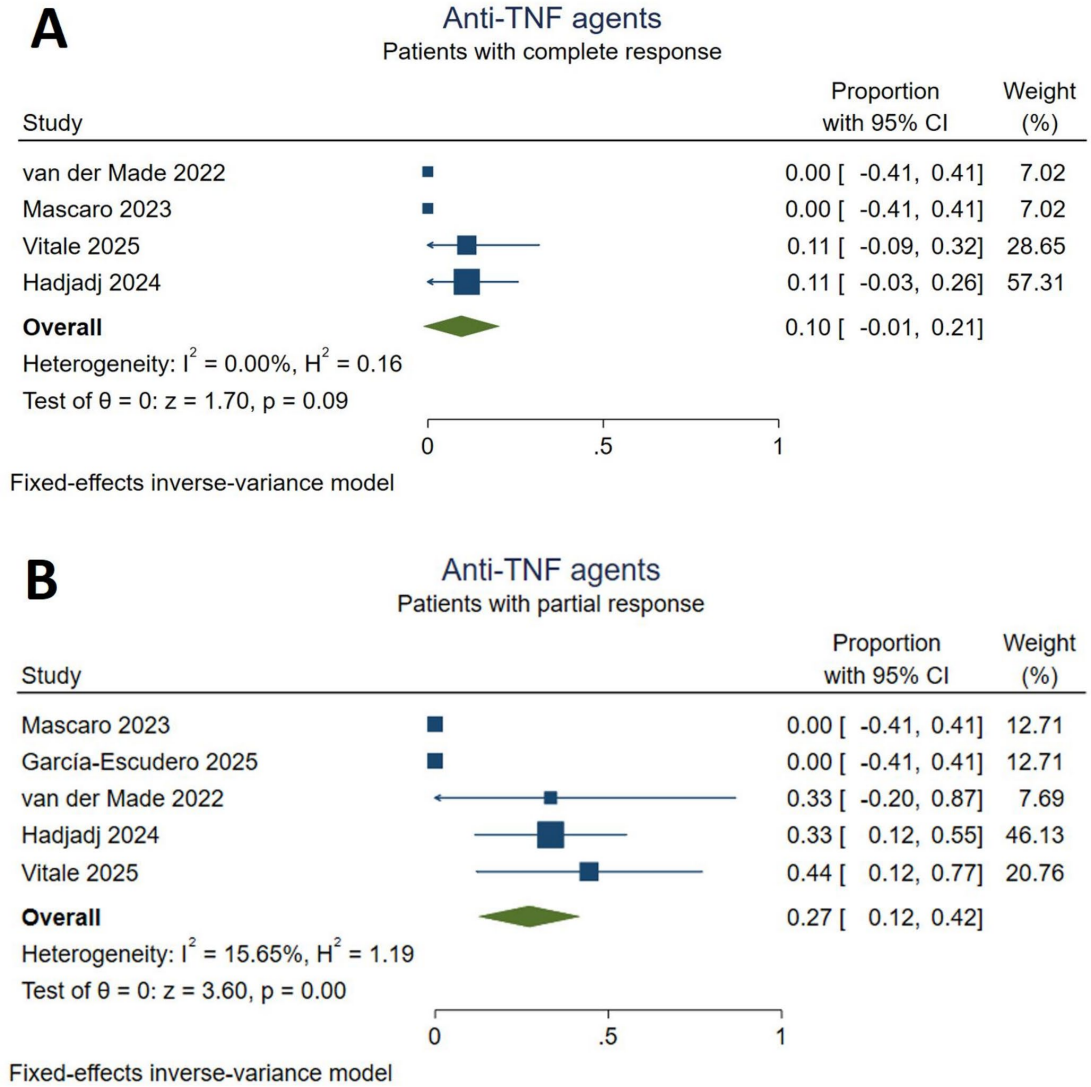
Response to anti-TNF treatment. **A**: complete responders, **B**: partial responders

Treatment with anti-TNF agents were received by 36 (9.8%) patients. The included anti-TNF agents were infliximab (*n* = 14), adalimumab (*n* = 13), and etanercept (*n* = 7). Five studies [17, 18, 26, 30, 32] evaluated the overall efficacy and safety of anti-TNF agents. According to the meta-analysis, anti-TNF agents did not significantly increase the number of complete responders [10%, 95% CI (-0.01,0.21)]. The proportion of patients who partially responded to anti-TNF treatment was 27% [95% CI (0.12,0.42)]. The statistical heterogeneity was non-significant for both response outcomes (I^2^ = 0% and I^2^ = 15.65%, Fig. 6). Infections (18 reported events) and venous thromboembolisms (10 reported events) were the most commonly observed adverse events. Death and malignancy were also reported during anti-TNF therapy (*Supplementary Table S3).*

## Discussion

To our knowledge, this is the first systematic review that includes a meta-analysis evaluating multiple treatment modalities for the management of VEXAS syndrome. This study synthesized data from 16 studies of varying designs and a wide range of interventions, including 367 patients with confirmed diagnoses of VEXAS syndrome from 12 different countries. The resulting cohort demonstrated a high level of comprehensiveness and diversity, offering a robust framework for understanding this recently identified and rare condition. Outcomes of our systematic review and meta-analysis indicated that azacitidine and JAK inhibitors were beneficial in achieving a complete treatment response in a significant proportion of patients according to our response criteria. Although not as effective as azacitidine and JAK inhibitors, IL-6 inhibitors were also found to be effective in achieving partial responses. Anti-IL-1 and anti-TNF agents demonstrated poor efficacy in the management of patients with VEXAS syndrome, compared with other interventions. Adverse events were common throughout all treatment options and serious adverse events were also reported with lower prevalence. Our findings indicated that patients who received any of the studied interventions exhibited a notably elevated risk of cardiovascular adverse events and infections.

Azacitidine is an antineoplastic agent, primarily used in the treatment of hematological malignancies including MDS [34]. The drug was shown to be a potential treatment option for patients with VEXAS syndrome with concomitant MDS after its demonstration of effectiveness in a prospective phase 2 trial [35]. A recent study suggested that azacitidine may facilitate long-term clinical remission, which can be sustained even after treatment cessation as a result of the eradication of mutated variant alleles [23]. Findings of a recent systematic review by Boyadzhieva et al. indicated that azacitidine was a frequently administered and promising treatment option for VEXAS syndrome [36]. Taking into account the preexisting knowledge and the findings of our study, azacitidine may represent a suitable therapeutic option for patients with VEXAS syndrome and associated MDS. Additionally, the study by Álamo et al. demonstrated that hypomethylating agents led to overall clinical improvement and normalization of hematological and inflammatory markers by significantly reducing the VAF of UBA1 [33]. These findings suggest that hypomethylating agents may be effective in the treatment of VEXAS syndrome, regardless of the presence of MDS, while highlighting the need for further evidence.

JAK inhibitors are a class of targeted therapies that inhibit the activity of the JAK/STAT pathway, which has an essential role in cytokine signaling. These inhibitors are utilized in the treatment of various autoinflammatory conditions and myeloproliferative neoplasms [37, 38]. A retrospective multicenter study found that JAK inhibitors were generally effective in the management of VEXAS syndrome, with ruxolitinib demonstrating superior efficacy compared to other JAK inhibitors [10]. The systematic review by Boyadzhieva et al. also reported the superiority of ruxolitinib and highlighted the increased risk of venous thromboembolism associated with JAK inhibitor therapy. Bindoli et al. performed a case-based review focusing on JAK inhibitor treatment for VEXAS syndrome, concluding that JAK inhibitors are effective and suggesting them as a first-line option [36]. They attributed the higher effectiveness of ruxolitinib to its specificity for JAK1 and JAK2, a viewpoint with which we agree [39]. In our study, we were unable to assess and compare each JAK inhibitor alternative since the interested outcomes were not individually reported. However, the outcomes of our systematic review suggest that JAK inhibitors may represent a viable treatment option for VEXAS syndrome.

IL-6 targeting therapies have been used in the treatment of autoinflammatory conditions and may provide improvements in suppressing inflammation in patients with VEXAS syndrome [40]. According to the outcomes of this study, tocilizumab may provide a partial response in a substantial proportion of patients with VEXAS syndrome; however, its efficacy in achieving a complete response was found to be limited. We propose that the underlying reason for this limited effectiveness may stem from a lack of influence from a hematological perspective. Nevertheless, IL-6 inhibitors may be administered to patients with high inflammatory activity and no hematological involvement. We found that anti-IL-1 and anti-TNF agents were ineffective in achieving a desirable response in patients with VEXAS syndrome and should not be prioritized in the treatment strategy.

We believe that the high adverse event profile observed in patients with VEXAS syndrome in our study can be interpreted from two perspectives. Firstly, the nature of the disease, which primarily affects individuals over the age of seventy, may directly influence their susceptibility to adverse events. The observed increase in cardiovascular adverse events in our study may be associated with this factor. On the other hand, the known severe side effect profiles of the studied interventions may also contribute to this outcome. The immunosuppressive characteristics of most of the drugs examined could explain the increased risk of infections. Regardless, physicians should be aware that these patients carry a high risk of adverse events during treatment, and they should be closely monitored throughout the duration of therapy.

This study had several limitations. First and the most important of them was the lack of prospective clinical trials and a limited number of cohort studies. A significant amount of the included studies were case-series and conference abstracts, that carry a higher risk of involving deficiencies in reporting. To minimize the impact of case series studies with a small number of patients on the meta-analysis, we initially employed the fixed-effects model. Due to the absence of a consensus on treatment response criteria for VEXAS syndrome, there may be discrepancies between the studies included and the response criteria we established. Other limitations included variations in response assessment time points, differences in medication dosing protocols, and the potential impact of concomitant prednisone dosing on treatment response. As the included studies did not specifically report outcomes for subgroups of drug classes and patients with MDS, separate statistical analyses could not be performed for these subgroups. The exact prevalence was not specifically reported for each adverse event in a sufficient amount of studies; therefore, we were unable to conduct a meta-analysis for safety outcomes. Additionally, some outcomes of our study exhibited considerable statistical heterogeneity.

This was the first systematic review and meta-analysis to assess the efficacy and safety of non-invasive treatment options for VEXAS syndrome. Outcomes of the study suggest that azacitidine and JAK inhibitors may be first-line treatment alternatives for patients with VEXAS syndrome and concomitant MDS. IL-6 inhibitors may provide improvements to the inflammatory aspect of the disease. Anti-IL-1 and anti-TNF agents should not be considered until treatment with the aforementioned alternatives has failed. The elevated risk of infections and cardiovascular adverse events should not be overlooked, and these patients must be closely monitored throughout the course of treatment. The limitations of our study, primarily the absence of prospective clinical trials, should not be overlooked when drawing conclusions from the findings of this work. Further clinical trials with a higher quality of design are needed to expand our knowledge about this topic.

## Electronic supplementary material

Below is the link to the electronic supplementary material.

Supplementary Material 1

## Data Availability

No datasets were generated or analysed during the current study.
