# Supplementary material for: Emerging treatment approaches for VEXAS syndrome: a systematic review and meta-analysis
Source: Ann Hematol. 2025 Apr 27;104(5):2617–30. doi: 10.1007/s00277-025-06382-2 (PMC12141402; doi:10.1007/s00277-025-06382-2)

**Supplementary table S1:** Quality assessment of the included studies according to NIH Quality Assessment Tool for Observational Cohort and Cross-Sectional Studies............................................... 2

**Supplementary table S2:** Quality assessment of the included studies according to NIH Quality Assessment Tool for Case Series Studies................................................................................................ 3

**Supplementary table S3:** Safety profile of the included interventions.................................................. 4

**Supplementary figure S1:** Random-effects meta-analysis of complete response outcome for azacitidine............................................................................................................................................... 5

**Supplementary figure S2:** Random-effects meta-analysis of partial response outcome for IL-6 inhibitors................................................................................................................................................. 6

**Supplementary figure S3:** Sensitivity analysis with leave-one-out method of partial response outcome for IL-6 inhibitors..................................................................................................................... 7

**Supplementary figure S4:** Random-effects meta-analysis of partial response outcome for anti-IL-1 agents..................................................................................................................................................... 8

**Supplementary figure S5:** Sensitivity analysis with leave-one-out method of partial response outcome for anti-IL-1 agents.................................................................................................................. 9

**Supplementary table S1:** Quality assessment of the included studies according to NIH Quality Assessment Tool for Observational Cohort and Cross-Sectional Studies

|  | Q1 | Q2 | Q3 | Q4 | Q5 | Q6 | Q7 | Q8 | Q9 | Q10 | Q11 | Q12 | Q13 | Q14 | Overall | |
| --- | --- | --- | --- | --- | --- | --- | --- | --- | --- | --- | --- | --- | --- | --- | --- | --- |
| Hadjadj 2024 | Yes | Yes | Yes | Yes | No | Yes | Yes | Yes | Yes | Yes | Yes | No | less than %20 | Yes | Good |  |
| Mascaro 2023 | Yes | Yes | Yes | Yes | No | Yes | Yes | No | No | No | No | No | less than %20 | Yes | Fair |  |
| Trikha 2024 | Yes | Yes | Yes | Yes | No | Yes | Yes | No | No | No | No | No | less than %20 | No | Fair |  |
| Jachiet 2024 | Yes | Yes | Yes | Yes | No | Yes | Yes | No | No | No | Yes | No | less than %20 | No | Poor |  |
| Kirino 2024 | Yes | Yes | Yes | Yes | No | Yes | No | Yes | Yes | No | Yes | No | less than %20 | Yes | Good |  |
| Garcia-Escudero 2025 | Yes | Yes | Yes | Yes | No | Yes | Yes | No | No | No | No | No | Less than %20 | Yes | Poor |  |
| Vitale 2025 | Yes | Yes | Yes | Yes | No | Yes | Yes | Yes | Yes | No | Yes | No | Less than %20 | Yes | Good |  |

**Source:** https://www.nhlbi.nih.gov/health-topics/study-quality-assessment-tools

**Supplementary table S2:** Quality assessment of the included studies according to NIH Quality Assessment Tool for Case Series Studies

| Study Label | Q1 | Q2 | Q3 | Q4 | Q5 | Q6 | Q7 | Q8 | Q9 | Overall |
| --- | --- | --- | --- | --- | --- | --- | --- | --- | --- | --- |
| Campochiaro 2022 | Yes | Yes | NR | Yes | Yes | No | Yes | No | Yes | Good |
| Pinto 2023 | Yes | Yes | NR | Yes | No | No | No | Yes | No | Fair |
| Islam 2022 | Yes | Yes | Yes | No | No | No | Yes | No | Yes | Poor |
| Williams 2024 | Yes | Yes | NR | Yes | No | No | Yes | No | Yes | Fair |
| Aalbers 2024 | Yes | Yes | Yes | Yes | Yes | No | Yes | Yes | Yes | Good |
| van der Made 2022 | Yes | Yes | NR | Yes | Yes | No | Yes | Yes | Yes | Good |
| Salehi 2023 | Yes | Yes | NR | Yes | Yes | No | No | Yes | Yes | Good |
| Alamo 2025 | Yes | No | NR | Yes | Yes | No | Yes | Yes | Yes | Good |

**Source:** https://www.nhlbi.nih.gov/health-topics/study-quality-assessment-tools

**Supplementary table S3:** Safety profile of the included interventions

| Treatment | Reported adverse events (number of events, %) | Reported serious adverse events (number of events, %) |
| --- | --- | --- |
| JAK inhibitors | Cytopenia(20, 13)  Infection (9, 11)  Thrombosis (5, 3)  Gland swelling (2, 1)  Insomnia(1,0.6) | Cardiovascular (4, 7)  Pneumonia (1,2)  Sepsis (1,2)  Gut Perforation (1,2) |
| IL-6 inhibitors | Cytopenia (24, 15)  Infection (22, 11)  Thrombosis (7, 4)  Hepatitis (3, 2)  Atopic Reaction (2,1) | Death (4,7)  Cardiovascular (4,7)  Cancer (4,7)  Pneumonia (2,4)  ARDS (1,2) |
| Anti-IL-1 agents | Cytopenia (8,5)  Oral ulcers (1,0.6)  Infection (1,0.6) | Severe ISR (9,16)  Cardiovascular (1,2) |
| Anti-TNF | Infection (18,11)  Venous thromboembolism (10,6)  Herpes zoster (4,2)  COVID-19 pneumonia (4,2)  Skin ulceration (2,1)  Pancytopenia (1,0.6)  Leukopenia (1,0.6)  Celulitis (1,0.6)  Recurrent phlebitis(1,0.6)  Small vessel vasculitis (1,0.6) | Cancer (6,10)  Death (6,10)  Severe neutropenia (2,4)  Bowel perforation (2,4)  Nocardia infection (1,2)  P.Jirovecii pneumonia (1,2)  Legionella infection (1,2)  Heart and Kidney Failure (1,2)  Sepsis (1,2) |
| Azacitidine | Infections (9,6)  Oral ulcers (1,0.6)  Fatigue (1,0,6)  Neutropenia (1,0.6) | Death (9,16) |

**Supplementary figure S1:** Random-effects meta-analysis of complete response outcome for azacitidine


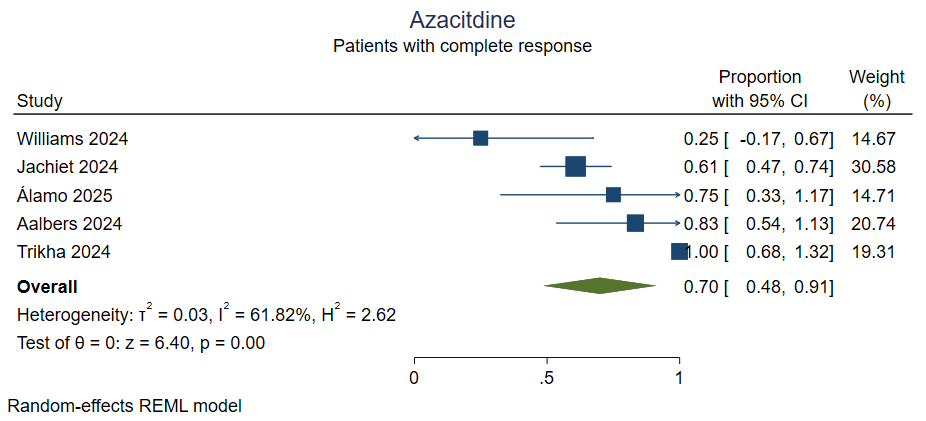


**Supplementary figure S2:** Random-effects meta-analysis of partial response outcome for IL-6 inhibitors

**
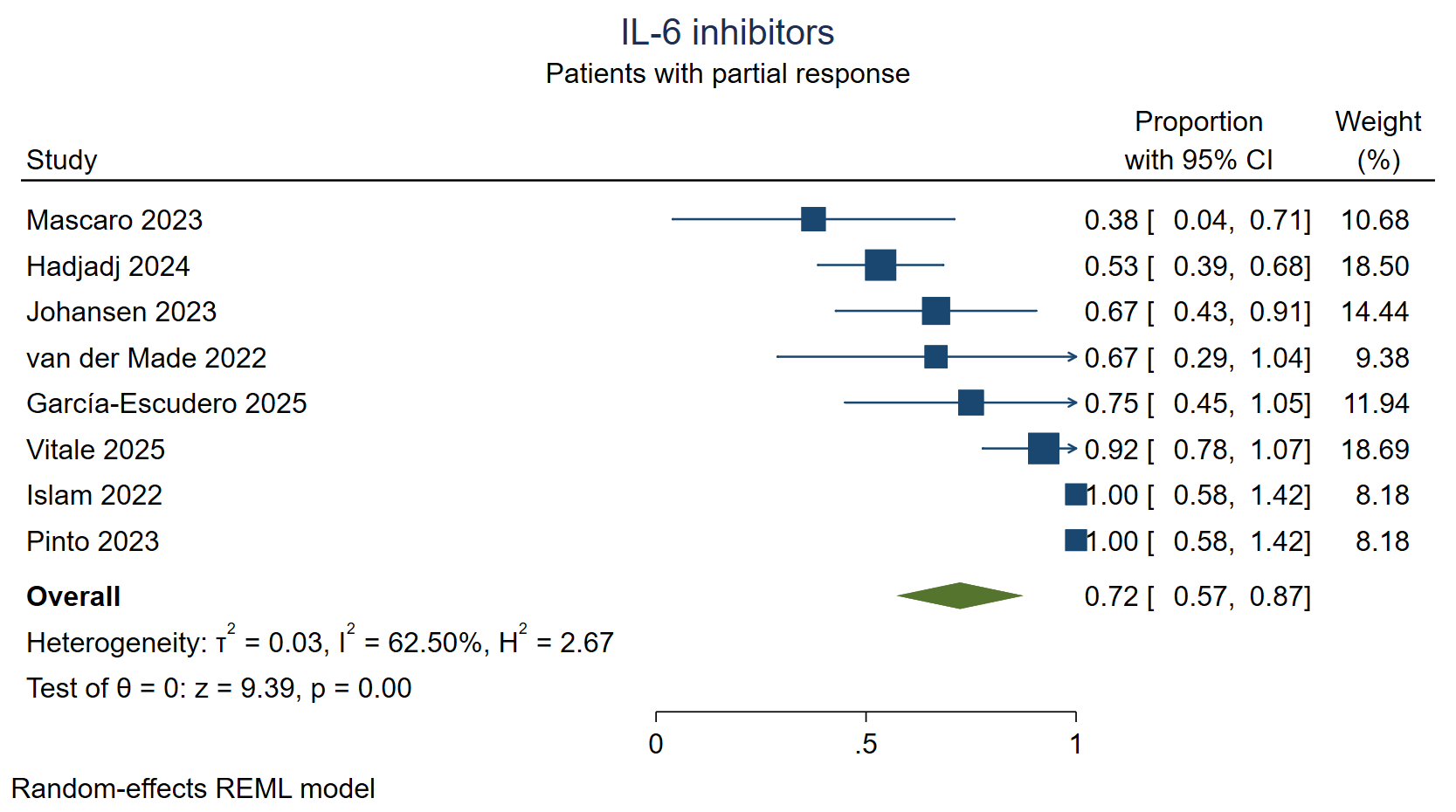
**

**Supplementary figure S3:** Sensitivity analysis with leave-one-out method of partial response outcome for IL-6 inhibitors


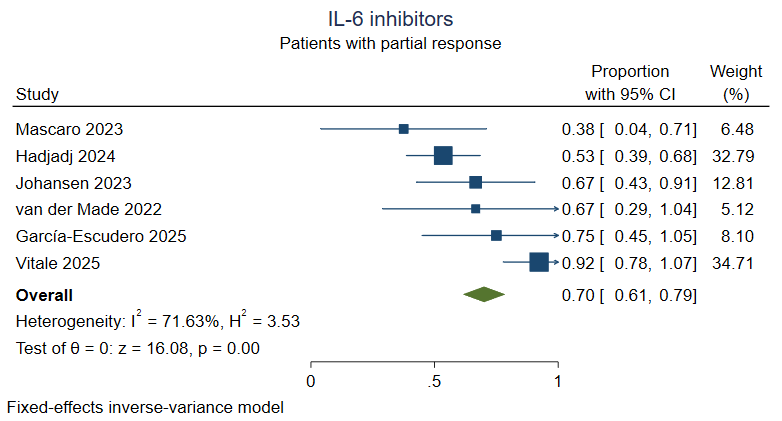


**Supplementary figure S4:** Random-effects meta-analysis of partial response outcome for anti-IL-1 agents


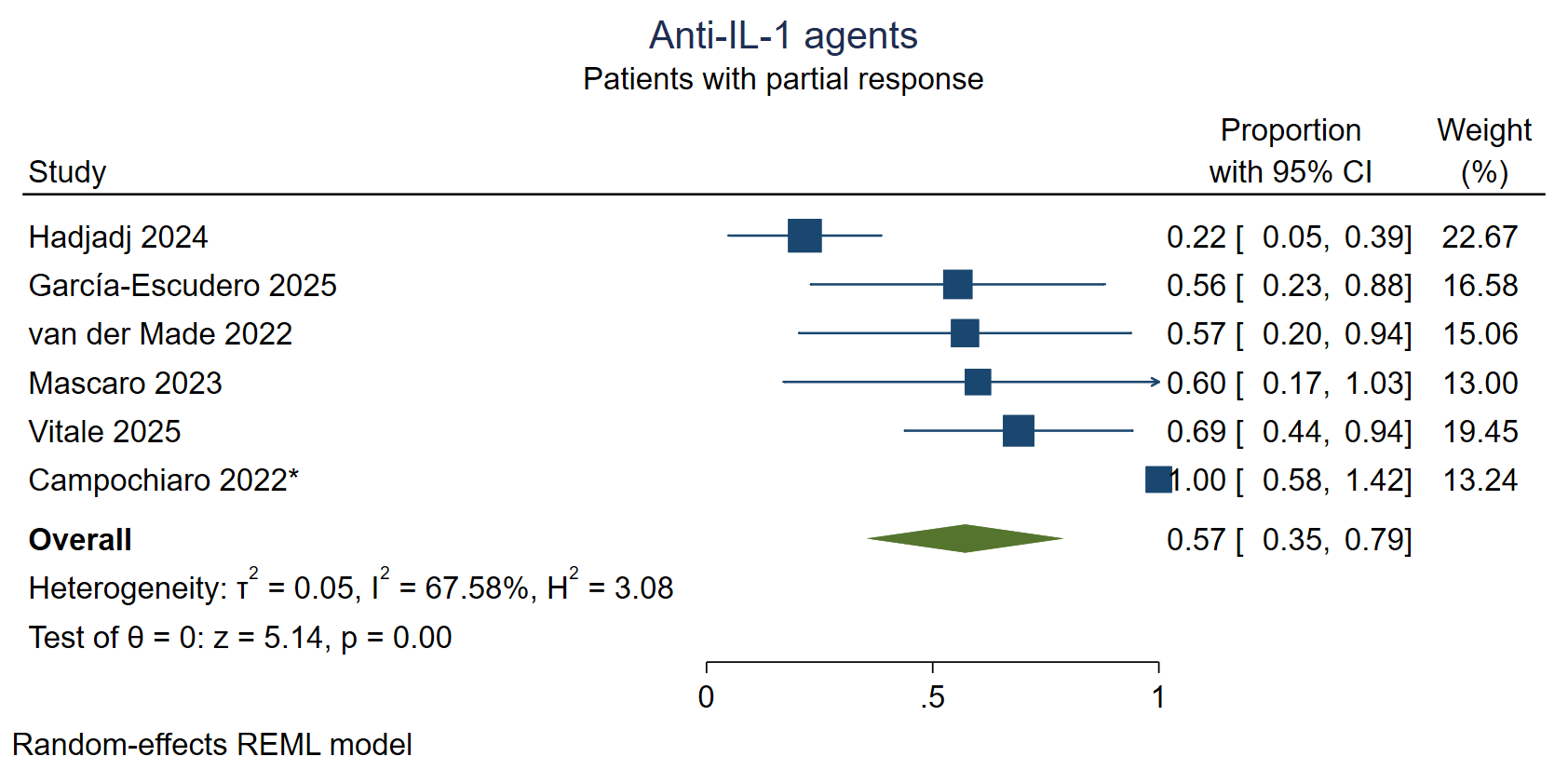


**Supplementary figure S5:** Sensitivity analysis with leave-one-out method of partial response outcome for anti-IL-1 agents


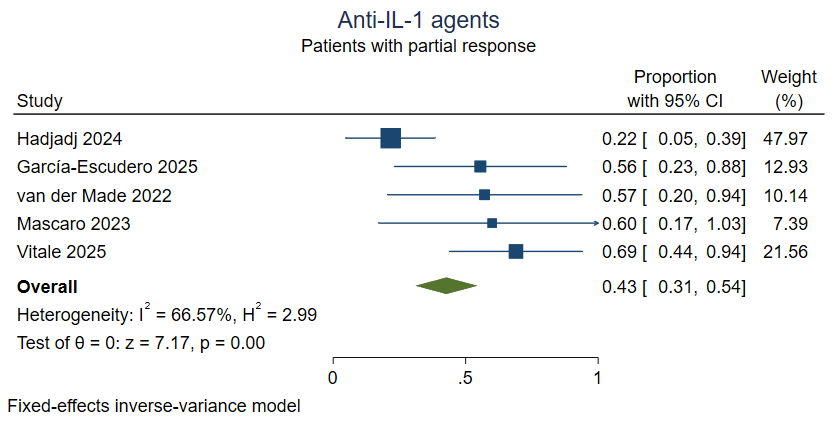

Supplement: Supplementary file 1 — Supplementary Material 1 [file 277_2025_6382_MOESM1_ESM.docx]
